# Defining competency frameworks for undergraduate and graduate pharmacology education in Canada

**DOI:** 10.3389/fphar.2026.1952861

**Published:** 2026-10-28

**Authors:** Karim S. Ibrahim, Fatima Mraiche, Bradley L. Urquhart, John J. Leddy, Amy Todd, Dylan Burger, Fabiana A. Caetano Crowley

**Affiliations:** 1 Department of Cellular and Molecular Medicine, University of Ottawa, Ottawa, ON, Canada; 2 Department of Pharmacology and Toxicology, Alexandria University, Alexandria, Egypt; 3 Department of Pharmacology, Faculty of Medicine and Dentistry, College of Health Sciences, University of Alberta, Edmonton, AB, Canada; 4 Department of Physiology and Pharmacology, Western University, London, ON, Canada; 5 Department of Human Biosciences, Memorial University of Newfoundland, St. John’s, NL, Canada; 6 The Jones Family Kidney Research Centre, Inflammation and Chronic Disease Program, Ottawa Hospital Research Institute, Ottawa, ON, Canada

**Keywords:** competency framework, curriculum, Delphi method, graduate competencies, learning outcomes, mixed methods, pharmacology education

## Abstract

Pharmacology education in Canada spans multiple institutions and programs and is increasingly integrated with other disciplines. While this diversity is a strength, the absence of a national consensus on the core competencies required for graduation raises concerns about consistency across programs, accreditation, and potential gaps in knowledge and skills. To address these gaps, the Canadian Society of Pharmacology and Therapeutics (CSPT) launched an initiative to establish national competency frameworks for undergraduate and graduate pre-professional pharmacology programs. Using an exploratory sequential mixed methods design, an initial list of knowledge, skill, and attitude statements was developed through a literature review and iterative input from CSPT Education Committee members and participants in the 2024 CSPT Meeting Education Workshop. Semi-structured focus groups and interviews were then conducted with pharmacology professionals from the academic, public, and private sectors to explore their views on the core competencies for pharmacology graduates and to gather feedback on the initial list. Inductive content analysis revealed strong cross-sector agreement that the principles of drug action and therapeutic response are essential for pharmacology graduates. Academic sector participants emphasized pharmacokinetics and pharmacodynamics as core knowledge at both undergraduate and graduate levels. Private sector participants prioritized laboratory and regulatory competencies, and public sector participants emphasized interdisciplinary collaboration. A modified Delphi questionnaire with national panellists produced lists of 27 and 55 statements for the undergraduate and graduate levels, respectively. Consensus statements were then restructured into competencies, incorporating the level of knowledge or skill acquisition rated by panellists for each academic level. This yielded an undergraduate framework of 12 key and 32 enabling competencies, and a graduate framework of 17 key and 58 enabling competencies, representing the first consensus-based, level-specific competency frameworks for pharmacology programs in Canada. These frameworks will guide curriculum design, professional preparation, and pharmacology training needs.

## 1 Introduction

Pharmacology is an interdisciplinary science that integrates chemistry, physiology, and mathematics to understand how drugs interact with biological systems. The discipline incorporates experimental inquiry and clinical application, and this breadth is both a defining strength and a persistent challenge for educators tasked with identifying and teaching its core content (Engels, 2018; Guilding et al., 2024a). In Canada, pharmacology is taught across a wide range of programs, including biomedical science degrees, professional programs (pharmacy, medicine, nursing, dentistry, veterinary medicine), as well as postgraduate pharmacology programs (Crowley et al., 2025). These programs vary considerably in structure, scope, and degree of disciplinary integration. Some offer traditional major and minor degrees in pharmacology, while others provide combined degrees with related disciplines or applied degrees such as pharmaceutical sciences. For instance, among Canada’s U15 group of research-intensive universities, approximately 65% offer pharmacology as a stream or in combination with other undergraduate disciplines such as physiology, toxicology, or the health sciences. At the graduate level, approximately 75% of institutions embed pharmacology within combined or interdisciplinary frameworks (personal communication, June 2025). Pharmacology graduates typically pursue employment in universities, hospitals, pharmaceutical and biotech industries, private and public research organizations, government agencies, and consulting companies. The breadth of professional pathways raises the question of whether pharmacology graduates from varied program structures acquire comparable core competencies.

A competency is generally described as a group of related knowledge, skills, and attitudes that influence an individual’s performance and are assumed to be a prerequisite for adequate job functioning (Eraut, 1994; Baartman and de Bruijn, 2011). In higher education, a competency-based curriculum complements a concept-based curriculum by defining discipline-specific tasks that graduates must be able to perform using the concepts and skills learned throughout their training (Giddens, 2020). In Canada, competency frameworks are well established and extensively employed in regulated health professions. For example, the National Association of Pharmacy Regulatory Authorities (NAPRA) published entry-to-practice competencies for pharmacists and pharmacy technicians (NAPRA, 2024), and the Royal College of Physicians and Surgeons of Canada (RCPSC) defined competency standards for clinical pharmacologists and toxicologists through its CanMEDS framework (RCPSC, 2025). These frameworks, however, are developed for clinical practice and do not translate to non-clinical pharmacology programs. Internationally, there have been comparable efforts to define core knowledge and expected skills for undergraduate graduates in life sciences. The British Pharmacological Society (BPS) developed a United Kingdom core undergraduate pharmacology curriculum using a modified Delphi method (Wallace et al., 2021; The British Pharmacological Society, 2026), and the Physiology Majors Interest Group established a consensus professional skills framework for undergraduate physiology graduates through a national consultative process in the United States (French et al., 2020). At the level of disciplinary content, an initiative by the International Union of Basic and Clinical Pharmacology (IUPHAR) identified 24 global core concepts of pharmacology (White et al., 2023). Collectively, these efforts have been valuable in developing frameworks for regulated health professions or comparable program structures in the United Kingdom or the United States. However, the distinct landscape of Canadian pharmacology education, along with the unique expectations and demands placed on graduates by Canadian employers, limits the direct application of these frameworks.

There is no national consensus on core competencies for pharmacology graduates in Canada. In the absence of a framework, teaching pharmacology across heterogeneous curricular models can weaken disciplinary coherence and render graduate competencies dependent on program structure and institutional context (Rangachari, 1997; Crowley et al., 2025). This pattern is not unique to pharmacology. For instance, competency development for undergraduate biology has been identified as a relatively under-addressed aspect of curricula despite its recognized importance to career success across STEM fields (Clemmons et al., 2020). Similarly, transferable professional skills expected of physiology graduates are not consistently embedded across undergraduate programs (French et al., 2020). Employer expectations further compound this challenge with the consistently reported gaps between the skills new graduates demonstrate and those required in professional settings (Sinche et al., 2017; Hart Research Associates, 2018; Nelson and Yang, 2025). Whether comparable gaps exist in Canadian pharmacology trainees remains largely unexplored, highlighting the need for a framework that addresses such gaps and specifies performance criteria for graduates employed across the different professional sectors.

To address these gaps, the Canadian Society of Pharmacology and Therapeutics (CSPT) launched a national initiative to establish national competency frameworks for undergraduate and graduate pharmacology programs. In this study, we conducted a literature review and consultations to support the development of a list of knowledge, skill, and attitude statements. Semi-structured interviews and focus groups were then conducted with pharmacology professionals from academic, private, and public sectors to refine the proposed list. This was followed by a modified Delphi method with a national panel of pharmacology professionals across different sectors and recent pharmacology graduates to reach consensus on core competency statements. The competencies produced through this process represent the first consensus-based frameworks defining what Canadian pharmacology graduates should know and be able to do at the undergraduate and graduate levels.

## 2 Methods

### 2.1 Framework development and study design

An initial list of knowledge-, skill-, and attitude-based statements for the pharmacology curriculum was developed through reviewing published pharmacology curricula reports as well as consensus literature in relevant disciplines. We prioritized sources that described transparent statement generation and expert consensus processes. This included the BPS undergraduate pharmacology core curriculum (Wallace et al., 2021), NAPRA entry-to-practice competencies for pharmacists and pharmacy technicians (NAPRA, 2024), the RCPSC competency framework for clinical pharmacologists and toxicologists (RCPSC, 2025), earlier Delphi-based curriculum work in clinical pharmacology and therapeutics (Walley and Webb, 1997), and a professional skills list for undergraduate physiology majors developed through the Physiology Majors Interest Group (French et al., 2020). The literature review did not follow a systematic, database-structured search, as it was intended to generate a list of candidate statements to be further reviewed and mapped to Canadian pharmacology programs. The CSPT Education Committee, comprising pharmacology educators from different Canadian universities, refined the list through iterative review cycles focused on statement clarity, scope, and fit for Canadian programs. Edits and rewording rationales from each cycle were tracked in a version log. The Education Committee also facilitated an educators’ workshop at the 2024 CSPT Meeting to review the list and gather members’ input. The initial list comprised 76 statements: 32 knowledge-based, 27 skill-based, and 17 attitude-based statements (Supplementary File S1). The list’s coverage was subsequently expanded through analysis of the Phase 1 focus groups and interviews described below.

Following the development of the initial list, we employed an exploratory sequential mixed methods approach (Figure 1; Torrance, 2012). Phase 1 involved semi-structured focus groups and one-on-one interviews. Transcripts were analyzed to refine, expand, and contextualize the initial list. Specifically, of the 76 initial statements, 30 were merged into consolidated statements, 11 were split, 13 were removed, and 22 were retained with or without rewording. The merged, split, and retained statements formed 50 stakeholder-amended statements. A further 13 statements were newly generated directly from focus group and interview data. Supplementary File S1 outlines the changes applied to each initial statement and traces the development of the 63 stakeholder-amended statements. We then reviewed the resultant stakeholder-amended statements for content validity and comprehensiveness. In Phase 2, we administered a modified Delphi questionnaire to achieve structured consensus with iterative refinement across two rounds (Fetters et al., 2013; Diamond et al., 2014). The study was approved by the University of Ottawa Research Ethics Board (H-02-25–11127). All study activities occurred online to support participation across Canada and different time zones.

**FIGURE 1:**
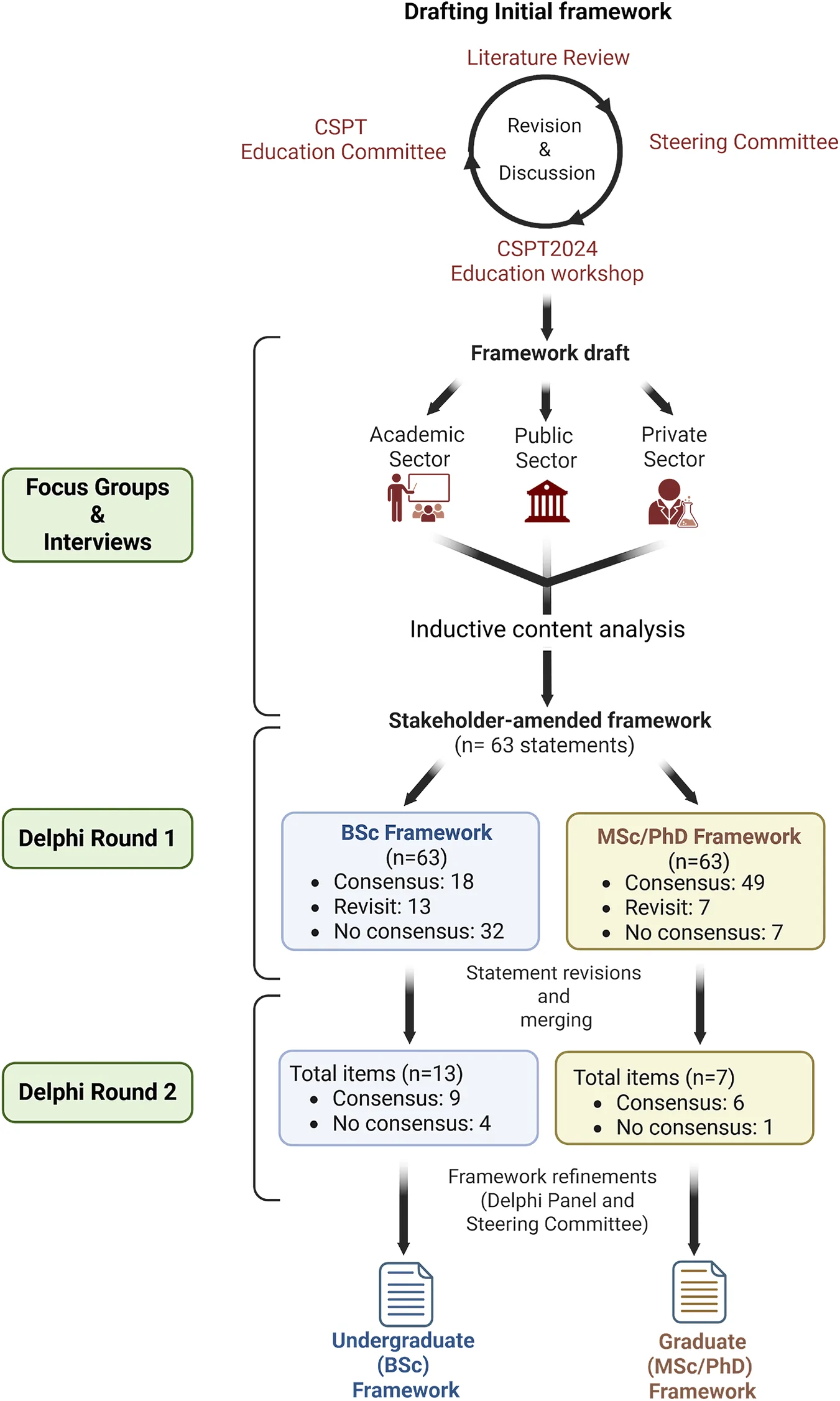
Summary of mixed methods study design used to develop pharmacology core competencies. An initial list of knowledge, skills, and attitude statements was produced through a literature review and iterative deliberation between the Canadian Society of Pharmacology and Therapeutics (CSPT) Education Committee, pharmacology educators at the 2024 CSPT Meeting Education Workshop, and the steering committee for this study. Focus groups and interviews were conducted with pharmacology professionals from academic, public, and private sectors to solicit feedback which informed a stakeholder-amended framework comprising 63 statements. In Delphi Round 1, panellists rated statements separately for undergraduate and graduate programs. Statements not reaching consensus were excluded or revised before Delphi Round 2. Final framework refinements yielded undergraduate (BSc or equivalent) and graduate (MSc/PhD) frameworks. Created in BioRender. Ibrahim, K. (2026) https://BioRender.com/m7h5u2.

### 2.2 Eligibility and participant recruitment

Eligible participants were at least 18 years old and proficient in English, and met at least one of the following criteria: (1) at least 2 years of experience in academia, defined as holding a graduate or professional degree and being affiliated with a Canadian academic pharmacology program or institution, including, but not limited to, roles such as instructor, lecturer, professor, program director, and scientist; (2) recent graduation from a Canadian pharmacology program within the past 2 years, with a minimum of a bachelor’s degree; (3) at least 2 years of experience in the public sector with experience in pharmacology, including, but not limited to, roles such as scientific evaluator, clinical evaluator, or policy analyst; or (4) at least 2 years of experience in the pharmaceutical/biotechnology industry, including, but not limited to, roles such as research and development scientist, medical science liaison, or quality control analyst. Recruitment targeted a minimum of 20 participants, with a minimum of five participants per area of expertise. This number is sufficient for statistical comparisons while maintaining homogeneity of the Delphi panel (Delbecq et al., 1976; Malterud et al., 2016; Belton et al., 2019).

For the focus groups and interviews, a purposive sampling approach was followed to recruit participants with direct professional experience in pharmacology training and practice across the academic, public, and private sectors (Campbell et al., 2020). Targeted recruitment emails were circulated through CSPT networks across Canadian universities. Participants in Phase 1 were given the option to opt into the Delphi panel. Broader invitations after data collection were extended to recruit additional Delphi panellists and promote sector representation. This included two rounds of email invitations distributed through the CSPT mailing list, each containing a link to the study website and a link to the consent materials and study information. The study was also announced on CSPT’s website. Furthermore, members of CSPT’s Board of Directors were encouraged to forward the study within their respective departments and institutions.

### 2.3 Phase 1: focus groups and interviews

#### 2.3.1 Design

Semi-structured focus groups (5–6 participants) were employed to review and prioritize the knowledge, skills, or attitudes essential for pharmacology graduates. Focus groups with academic and public sector participants were conducted separately to encourage candid discussion within each professional group (Krueger and Casey, 2014). In addition, we employed semi-structured one-on-one interviews for private sector participants as proprietary interests might constrain open discussion in group settings. Participants from each of the three sectors were invited to participate in Phase 1 based on their direct professional experience in pharmacology training and practice. Recruitment met or exceeded the minimum of five participants for each sector. This was consistent with a sample size guided by the specific aims and scope of Phase 1 (Malterud et al., 2016). Each session comprised two segments. First, we introduced the study rationale and aims. Participants were then presented with this prompt: *“Please think of a minimum of 2–3 core knowledge, skills, or attitudes that you believe are essential for pharmacology students to possess at the undergraduate, MSc/PhD levels, or both*” (Supplementary Table S1). Participants were guided to consider a pharmacology-dependent role in their professional setting and to describe what a qualified candidate must be able to do on day one and within the first year in that position. Next, participants reviewed the initial list of statements described in Section 2.1 and provided feedback on coverage, gaps, and additional statements. Sessions were conducted as online focus groups and interviews via an encrypted, university-licensed Zoom account (Zoom Communications, Inc.), audio-recorded with permission, transcribed using Zoom’s transcription tool, and de-identified before analysis (Gray et al., 2020).

#### 2.3.2 Qualitative and quantitative analyses

Focus group and interview transcripts were analyzed using inductive content analysis (Hsieh and Shannon, 2005; Elo and Kyngäs, 2008). The transcripts were coded and analyzed in NVivo 15™ software (QSR International Pty Ltd., Version 15.2.2). Two researchers (K.S.I. and F.C.C.) independently read the transcripts and assigned codes to meaningful units of text that captured the manifest content of participants’ responses (Graneheim and Lundman, 2004; Hsieh and Shannon, 2005). Each code was a descriptive label for the concept expressed in a unit of text. Codes were iteratively compared within and across transcripts, and similar codes were grouped into broader categories through a repeated process of comparison and consolidation. Discrepancies in coding were resolved through discussion and consensus among the research team while maintaining an audit trail. Categories were then abstracted and qualitatively analyzed by identifying conceptual connections across categories (Elo and Kyngäs, 2008). A reference denoted the presence of a given code in a participant’s responses, regardless of the number of coded text segments. Category totals were calculated by summing references across the category’s constituent codes. To quantitatively estimate the contribution of each sector to category references, the number of references attributed to participants within each sector was divided by the total reference count for each category, expressed as a percentage.

### 2.4 Phase 2: Delphi consensus process

#### 2.4.1 Design

We used a modified Delphi method to obtain structured consensus on the stakeholder-amended list from Phase 1. Our approach departed from the classical Delphi format in terms of not employing an open-ended round (Round 0) in which panellists nominate items for consideration (Khodyakov et al., 2023). Our panellists also rated each statement on a second dimension, its intended degree of acquisition, reflecting the framework’s aim of specifying the proficiency level expected of each competency. The Delphi questionnaires were designed and administered via SurveyMonkey (SurveyMonkey Inc.; www.surveymonkey.com). We pilot-tested the questionnaire procedures and data collection workflow before proceeding to the first round to assess feasibility and clarity. Five academics completed the pilot questionnaire and provided feedback on the instructions, statements, and usability. Pilot feedback resulted in minor wording edits for clarity only.

Each round remained open for 2 weeks (Fan and Yan, 2010; Ha et al., 2025). We sent two reminder emails to nonresponders in each round. The first page of the questionnaire provided information on core competency definitions, study purpose, ethics approval, and oriented panellists on the expected review tasks in the round. Each statement was classified as knowledge-, skill-, or attitude-based before distribution. The first question required panellists to rate the importance of every competency for each academic level: undergraduate (BSc or equivalent) and graduate (MSc or PhD) levels. A four-point Likert scale was used: “not at all important”, “less important”, “very important”, “essential”. A “not applicable or cannot assess” option was also included. This question was set as mandatory to ensure complete datasets (Supplementary Table S1). Panellists were assigned identification numbers to track participation across rounds. Identifying information was stored separately from questionnaire data.

For knowledge- and skill-based statements, a second question was added to capture the intended degree of acquisition for each competency statement per academic level (Supplementary Table S1). We selected Bloom’s taxonomy for knowledge-based statements, a well-established taxonomy of cognitive processes (Anderson and Krathwohl, 2001), and the Dreyfus model for skill-based statements, a widely accepted model for skill acquisition in clinical practice and education (Benner, 2004; Dreyfus, 2004). Panellists were asked to assign a Bloom’s taxonomy level to knowledge-based statements for each academic level. We used the revised Bloom’s taxonomy levels: “remember”, “understand”, “apply”, “analyze”, “evaluate”, and “create” (Anderson and Krathwohl, 2001). For skill-based statements, panellists were asked to assign a degree of skill acquisition using an adapted Dreyfus model (Benner, 2004; Dreyfus, 2004). We used four stages: “novice”, “advanced beginner”, “competent”, and “proficient”. We removed the “expert” stage to match expected graduation levels (Yeung et al., 2023). An additional comment field was provided to capture suggestions for rewording, additions, deletions, and any concerns about each statement. For each statement, the median selection across panellists determined the level of acquisition used in the final wording of the competency statement. To support consistent application of the Dreyfus model, panellists were also provided with a supplemental reference file containing example skill-based competencies for each Dreyfus level.

#### 2.4.2 Consensus thresholds

A minimum of two Delphi rounds is required to achieve consensus through iteration, a defining characteristic of the Delphi method (Diamond et al., 2014). For this study, a maximum of three rounds was planned, provided that consensus had not been reached on all statements after the second round. Inclusion thresholds were defined *a priori* and applied separately for each academic level: undergraduate and graduate levels. A statement was retained in the framework for a given academic level if ≥ 75% of panellists rated it as “very important” or “essential” at that level. The 75% inclusion threshold as percent agreement is a frequently reported midpoint threshold for Delphi consensus across published studies (Diamond et al., 2014). Statements that received an acceptance rate of 65%–74% were flagged for re-rating in a subsequent round, while statements receiving an acceptance rate below 65% were excluded with no further discussion. To account for unequal group sizes across the four Delphi panel groups (Academic, Public, Private, Recent Graduates), we calculated the acceptance rate within each sector separately and averaged the four values, assigning each sector equal weight in the final consensus calculation. This approach was adopted to mitigate dominance of the larger academic group over the consensus calculation and to preserve the multi-sector intent of the framework (Venda Nova et al., 2023; Williams et al., 2024). Delphi ratings were summarized using descriptive statistics, and open-text feedback was analyzed to identify issues of clarity and scope and was used to revise statements before subsequent rounds. All statement-level actions were recorded across rounds, including rewording, merging, splitting, and removal.

#### 2.4.3 Statistical analysis

To examine Likert rating distribution differences across the four panel groups, a Kruskal–Wallis test followed by Dunn’s *post hoc* test was applied for undergraduate-level and graduate-level statements (Corder and Foreman, 2009; Tolsgaard et al., 2013). As a nonparametric test, the Kruskal–Wallis test accounts for unequal group sizes (Corder and Foreman, 2009). For each statement, the four panel groups were compared using their median ratings. A p-value less than 0.05 was considered statistically significant. These analyses were conducted in GraphPad Prism 8.

## 3 Results

Twenty-one participants completed Phase 1 of the study (Table 1). Participants were based across five provinces: Ontario, British Columbia, Alberta, Quebec, and Nova Scotia. The academic sector constituted the largest group (n = 11), followed by participants from the public sector (n = 5) and the private sector (n = 5). Academic focus group participants included Associate Deans of Education and Program Directors, as well as faculty with varying teaching loads. Most participants reported more than 10 years of professional experience in pharmacology-related fields. All 21 participants opted into the Delphi panel. Broader recruitment added additional panellists to a total of 34 participants. Of these, 29 participants completed Delphi Round 1, and 28 completed Delphi Round 2.

**TABLE 1:** Study participant demographics.

Variable	Focus groups/Interview sessions counts (%)	Delphi Round 1 counts (%)	Delphi Round 2 counts (%)
Total number of participants	21	29	28
Area of professional expertise^a^ Academia			
• Academic administrative leadership	4 (15)	3 (10)	3 (10)
• With ≥50% teaching load	5 (19)	9 (29)	9 (30)
• With <50% teaching load	8 (30)	8 (26)	7 (23)
Government/Provincial sector	5 (19)	3 (10)	3 (10)
Private sector	5 (19)	4 (13)	4 (13)
Recent pharmacology graduates	0 (0)	4 (13)	4 (13)
Years of experience in pharmacology			
0–5 years	1 (5)	4 (14)	4 (14)
6–10 years	2 (10)	4 (14)	4 (14)
11–20 years	9 (43)	12 (41)	12 (43)
20+ years	9 (43)	9 (31)	8 (29)
Gender			
Female	6 (29)	13 (45)	13 (46)
Male	15 (71)	16 (55)	15 (54)
Other	0 (0)	0 (0)	0 (0)
Prefer not to answer	0 (0)	0 (0)	0 (0)

^a^ Each number reflects how many individuals held expertise in that area. Some participants spanned more than one category.

Results are reported across four stages of framework development: the categories identified through inductive content analysis of the focus group and interview transcripts (Section 3.1), the stakeholder-amended statements administered in Delphi Round 1 (Section 3.2.1), the statements reaching Delphi consensus (Sections 3.2.1 and 3.2.2), and the final competency frameworks derived from consensus statements (Section 3.2.3).

### 3.1 Inductive content analysis

Inductive content analysis of the focus group and interview transcripts yielded 12 categories. The categories collectively characterized the knowledge, skills, and attitudes that participants identified as essential for pharmacology graduates at both academic levels (Figure 2; Supplementary Table S2). Nine of the 12 categories were referenced by participants in all three sectors (Figure 2). We quantitatively assessed the cross-sector distribution of category references by calculating each sector’s percentage share of the total references of the codes within each category (Figure 2). This distribution reflects each sector’s relative emphasis on each category (Sandelowski et al., 2009; Maxwell, 2010). Accounts from the academic sector focus groups generated the majority of references to drug action and therapeutic response (58%), scientific literacy skills (83%), and principles of life sciences (60%) (Figure 2). Scientific literacy skills and knowledge of principles of life sciences were identified by academic and private sector participants; neither category was raised by public sector participants (Figure 2). Private sector participants contributed to the majority of references on the methodological approaches and technical skills category (100%), including Good Laboratory Practice (GLP) and Good Manufacturing Practice (GMP), as well as drug discovery and regulation (70%) and interpersonal skills (59%). The accounts in the public sector focus groups generated the largest share of references on the translational and clinical pharmacology category (45%) (Figure 2). Principles of drug safety and professional identity and development were the categories that showed the most evenly distributed reference patterns (Figure 2). For the drug safety category, academic and public sector participants each contributed 40% of reference frequencies. For the professional identity category, private and public sector participants each contributed approximately 43% (Figure 2).

**FIGURE 2:**
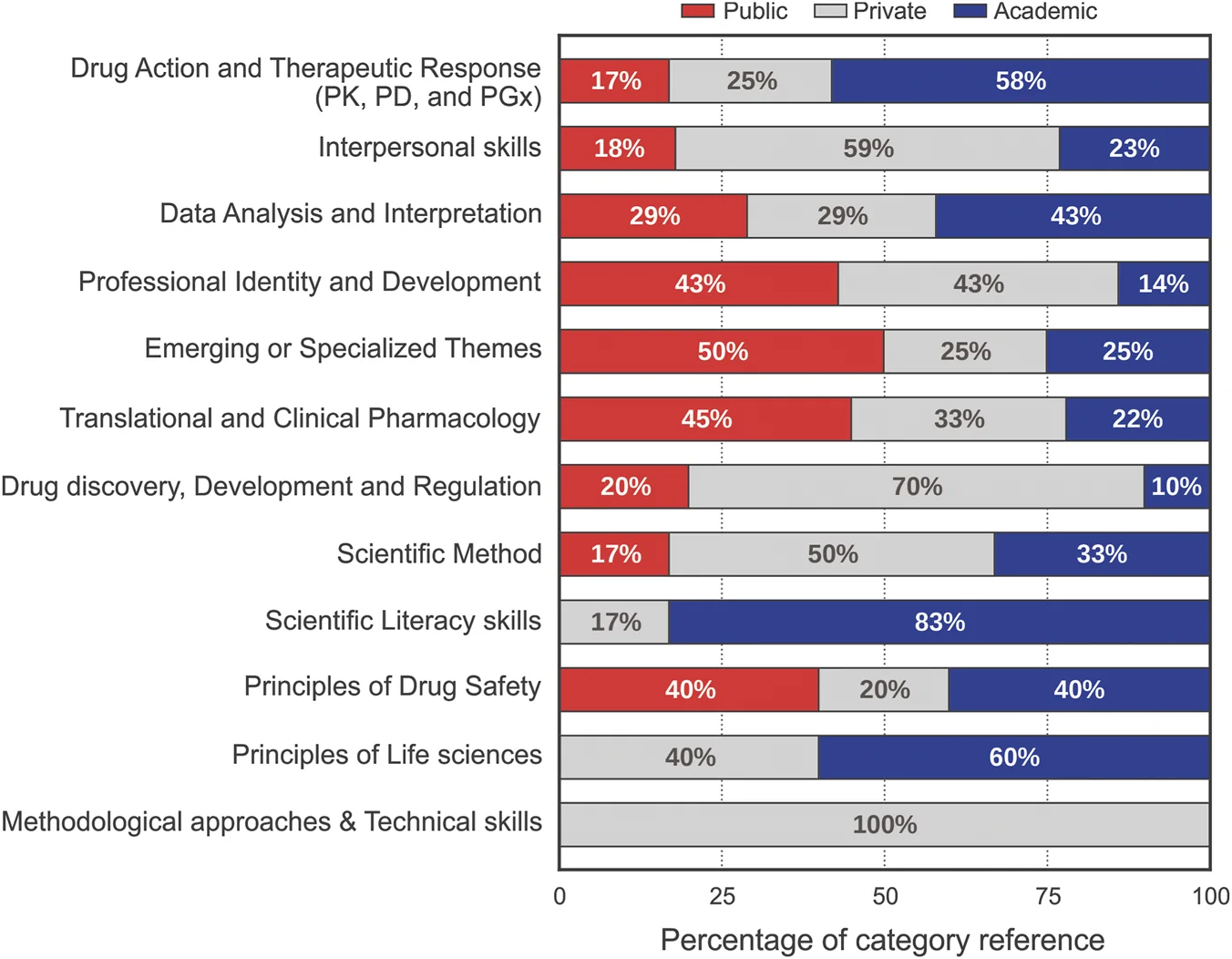
Cross-sector distribution of category references across focus groups and one-on-one interviews. Twelve categories were identified through inductive content analysis applied independently by two members of the research team. Bars represent each sector’s percentage share of the total references of the codes within each category, attributed to Public (red), Private (grey), and Academic (blue) sector participants. PK, pharmacokinetics; PD, pharmacodynamics; PGx, pharmacogenomics.

#### 3.1.1 Foundational knowledge as the basis for clinical and therapeutic application

The principles of drug action and therapeutic response was the most frequently referenced category that emerged across all three sectors and at both academic levels. Pharmacokinetic (PK) and pharmacodynamic (PD) concepts were consistently identified as essential foundational knowledge that is built on prerequisite life sciences knowledge, including cell biology, physiology, medicinal chemistry, and quantitative mathematics. A private sector participant described this expectation in terms of progressive biological understanding from the cellular to the systemic level: “*They [graduates] need to understand what a cell is, the basic anatomy, physiology of a cell, and the downstream signals that are within the cell*” (PV#5*).* Academic sector participants additionally identified pharmacogenomics as a basis for understanding interindividual variability in drug response. On the other hand, private sector participants emphasized applied integration of PK and PD knowledge with an expectation that graduates should be able to trace the compound’s systemic exposure through to its receptor-level response: “*So what I expect in the skills here that we can link the pharmacodynamics to the pharmacokinetics. For example, based on the knowledge of pharmacology, we will be able to understand when a compound has some exposure in the systemic circulation that will lead to an interaction with this target, with this receptor, but the response will be also modulated by the presence of the compound based on the pharmacokinetics*” (PV#5).

This continuity from foundational science to applied practice was evident in how participants in the public and academic sectors described translational and clinical pharmacology. A public sector participant emphasized the importance of pharmacology graduates extrapolating PK data from animal studies to inform first-in-human dose decisions: *“Having to think about what the kinetics study in animals might mean for kinetics in humans ... it is important in terms of deciding on the first human dose”* (PB#5). Another academic sector participant characterized the disconnect between fundamental pharmacology principles and clinical relevance as a particularly salient challenge for modern pharmacology education: “*We have clinical guidelines for drug use, and sometimes we do not make the connection between the fundamentals of pharmacology and clinical guidelines. And I think that applies to both graduates and undergraduates. I feel like that’s a modern pharmacology competency that we should be thinking about*” (AC#10).

Private sector participants applied this same connection between foundational knowledge and professional practice to drug discovery, development, and regulation. They drew a clear distinction between undergraduate and graduate expectations. For undergraduates, participants described an expectation of conceptual familiarity with how drugs move from discovery through clinical evaluation. For graduates, expectations extended to knowing the requirements of major regulatory bodies: “*They should have some knowledge about regulatory aspects of drug development, like what an IND is, what does the FDA require for approval of a drug? What are the different types of trials? These are going to be very important*” (PV#2).

#### 3.1.2 Development of independent scientific reasoning and research literacy

Academic and private sector participants emphasized the essential knowledge and skills for scientific inquiry and research competence, and stratified the expectations by the graduate training level. Critical appraisal of the primary literature was treated as a graduate-level competency, while an appreciation for evidence-based pharmacology was described as appropriate for undergraduates. One participant situated this expectation within the demands of a rapidly evolving field: “*This is probably a fairly obvious one for graduate students, being able to critically assess the biomedical literature; I think it is a key one. For undergraduates, critical assessment of the literature might be a bit much, but just recognizing that for a field like pharmacology, where it is a live field, unlike some others where there’s not a whole lot of change. The biomedical literature is the source of up-to-date information on emerging drug targets. For undergrads, just the recognition of the value of the literature*” (AC#10). Academic sector participants further articulated a graduate-level expectation in formulating and testing original hypotheses: “*At the graduate level, it is very important for them to be able to develop a clear hypothesis based on rationale that they get from the current literature, and even more so, the best ways to address that hypothesis experimentally*” (AC#11). Private sector participants extended this emphasis by noting that effective scientific inquiry requires fluency across a range of analytical and experimental approaches: “*You really want to have a diverse toolkit to really know how to interrogate scientific questions*” (PV#1). This extended to the development and qualification of fit-for-purpose bioanalytical assays as a graduate-level expectation: “*How do you actually develop an assay for testing something related to your drug? How do you qualify it? How do you validate this assay?*” (PV#2). Furthermore, documentation was identified as a core professional task that academic training programs rarely teach explicitly: “*Something that’s quite important in industry is documentation. Starting to teach just the proper way to make data entries in a lab book: not erasing anything, marking only the important things, and adding initials. I think that’s important*” (PV#3).

Data analysis and interpretation also emerged as a priority competency across all three sectors, with a distinction in expectations between undergraduate and graduate levels. At the undergraduate level, participants underscored the importance of knowledge and application of core statistics to pharmacological data. Private sector participants additionally raised proficiency in digital data handling and statistical software as increasingly expected given the growing volume of computational and omics-based data in pharmacological research. A public sector participant described clinical statistical literacy as a desired graduate-level skill: *“When you get to the graduate level, understanding statistical design for clinical trials. It does not matter if you have to do it, but just understanding how those analyses are different, and that can be almost the cherry on top: getting a graduate student in a pharmacology program that comes with a strong clinical statistical component. That would be great*” (PB#2).

#### 3.1.3 Professional formation and career readiness

Interpersonal skills and professional competencies were considered essential to graduate career readiness. Participants noted that these competencies are rarely addressed explicitly during academic training. One public sector participant described the range of interdisciplinarity expected in the profession and the need to develop these skills more systematically within pharmacology training: “*We’re starting to coordinate more with other disciplines and other groups-really being able to coordinate with the electron microscope expert, or even with someone in a different department. I think those are skills that we assume people pick up gradually, and some people are good or not, but I think they need to be more effectively taught and communicated*” (PB#1). Private sector participants further reinforced the notion and pointed to the necessity of cross-disciplinary collaboration in drug development pipelines: “*Because these drug development processes require input from different disciplines, different fields. That requires really people to work together a lot*” (PV#2). Furthermore, public sector participants emphasized adaptability and ethical responsibility as key components for graduates’ professional identity. One participant argued that training must cultivate flexibility before graduates enter practice: “*Pharmacology graduate needs to be adaptable, but it is much easier when those tools are built into training rather than hitting a professional career all of a sudden and having to retool all the time. That flexibility is essential*” (PB#2). Ethical responsibility was also framed as a public obligation: “*I think ethics is pretty important. So much of our work as scientists is about trust. People need to trust us, and what we’re saying is real. So, I think we can’t take that for granted*” (PB#1).

#### 3.1.4 Forward-looking knowledge and skills

All three sectors converged on forward-looking knowledge and skill domains that are increasingly relevant to pharmacology training but not consistently embedded in existing curricula. One academic sector participant noted the importance of understanding and mitigating drug toxicity in the environment. In addition, a public sector participant identified the accelerating shift from animal models to organ-on-a-chip platforms as a transition with direct implications for graduate training: “*There’s going to be an increasing shift, and it is coming very fast down the line, from whole animals to either organs on a chip, or increasingly complex organoids. And when you look at all of that, I think it is important that pharmacologists understand that*” (PB#5). A private sector participant raised programming proficiency as an emerging graduate-level expectation for efficiently managing the increasing volume of digital pharmacological data (PV#3).

### 3.2 Modified Delphi method

#### 3.2.1 Delphi Round 1

Round 1 comprised 63 stakeholder-amended statements refined from the initial list using focus group and interview data, organized into three domains: Knowledge (n = 30), Skills (n = 22), and Attitudes (n = 11). To account for unequal group sizes, consensus was calculated using a sector-weighted approach, as described in Section 2.4.2. A summary of Round 1 results by domain and academic level is shown in Figures 3, 4, and Table 2. Statements submitted to this round and the consensus outcomes are provided in Supplementary Table S3. At the undergraduate level, 18 of 63 statements (28.6%) reached consensus, 13 met thresholds to advance to Round 2, and 32 were excluded (Table 2). The five knowledge-based statements reaching undergraduate consensus addressed core PK and PD concepts, including mechanisms of drug action, dose-response, and concentration-time relationships. Statements concerning pharmacogenomics, social determinants of health, and equity in pharmacology showed the highest rates of disagreement among panellists (Supplementary Table S3). Six out of 22 skill-based statements and seven out of 11 attitude-based statements reached consensus at the undergraduate level (Table 2).

**FIGURE 3:**
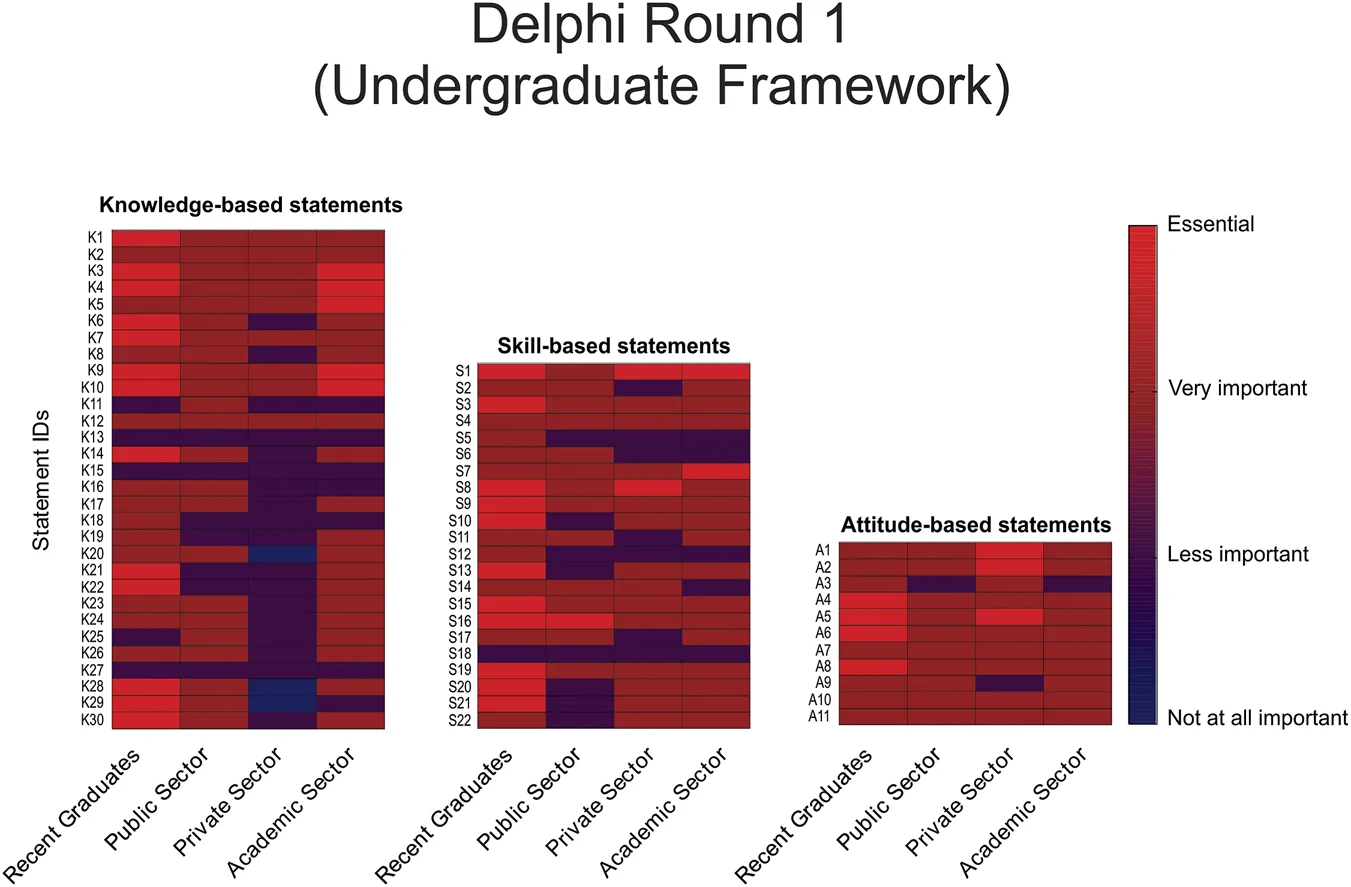
Heatmap of Likert importance ratings for undergraduate framework statements rated in Delphi Round 1. Statements are grouped by domain (30 knowledge-based, 22 skill-based, 11 attitude-based). Columns represent the four Delphi panel groups (Recent Graduates, Public Sector, Private Sector, Academic Sector). Cell color reflects the median rating on a four-point Likert scale ranging from “Not at all important” (dark blue) to “Essential” (bright red), as shown in the color bar.

**FIGURE 4:**
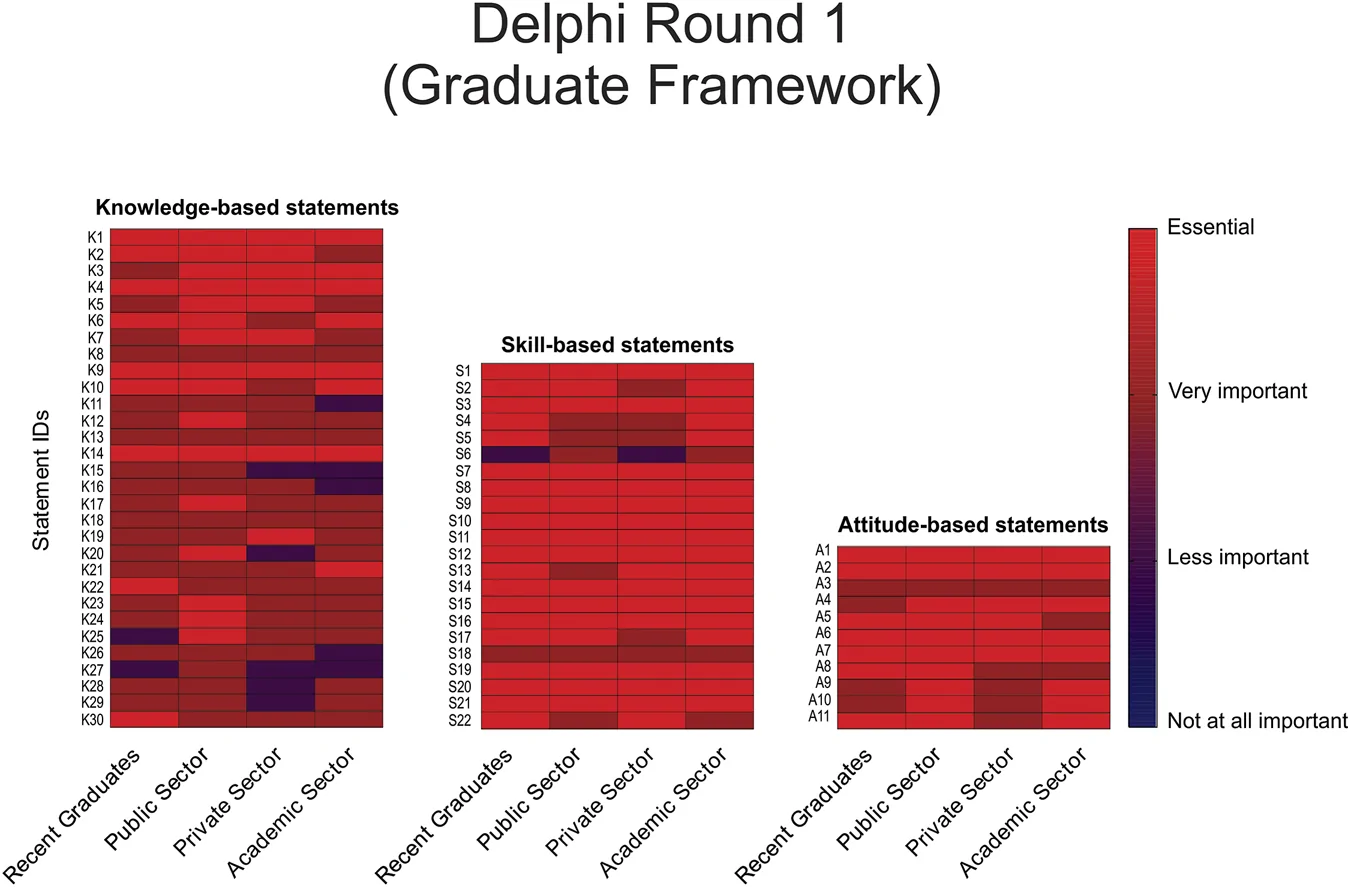
Heatmap of Likert importance ratings for the graduate framework statements rated in Delphi Round 1. Statements are grouped by domain (30 knowledge-based, 22 skill-based, 11 attitude-based). Columns represent the four Delphi panel groups (Recent Graduates, Public Sector, Private Sector, Academic Sector). Cell color reflects the median rating on a four-point Likert scale ranging from “Not at all important” (dark blue) to “Essential” (bright red), as shown in the color bar.

**TABLE 2:** Round 1 Delphi consensus outcomes by competency domain and academic level.

Domain	Undergraduate level (BSc or equivalent)			Graduate level (MSc or PhD)		
	Consensus	Revisit	No consensus	Consensus	Revisit	No consensus
Knowledge (n = 30)	5 (16.7%)	7 (23.3%)	18 (60%)	17 (56.7%)	7 (23.3%)	6 (20%)
Skills (n = 22)	6 (27.3%)	4 (18.2%)	12 (54.5%)	21 (95.5%)	0	1 (4.5%)
Attitudes (n = 11)	7 (63.6%)	2 (18.2%)	2 (18.2%)	11 (100%)	0	0
Total (n = 63)	18 (28.6%)	13 (20.6%)	32 (50.8%)	49 (77.8%)	7 (11.1%)	7 (11.1%)

At the graduate level, 49 of 63 statements (77.8%) reached consensus, seven required revisions, and seven were excluded. Disagreement was largely limited to the knowledge domain, where 17 of 30 statements (56.7%) reached consensus (Table 2). Disagreement among panellists was concentrated in knowledge-based statements addressing principles of toxicology, sustainability in pharmaceutical practice, and equity-framed content on Indigenous traditional medicine (Supplementary Table S3). The seven knowledge-based statements advancing to Round 2 addressed more specialized or context-specific content, including advanced pharmacokinetics, social determinants of health, medication safety, equitable access to medicines, and equity in research representation (Supplementary Table S3).

We applied a Kruskal–Wallis test to characterize differences in median Likert ratings among the Delphi groups, independently of the pre-specified consensus thresholds described in Section 2.4.2. For the undergraduate statements, there was a significant difference among groups (*H* = 42.76, *p* < 0.0001; Figure 3). Dunn’s *post hoc* comparisons showed that the recent graduates group rated undergraduate statements significantly higher than private (mean rank difference = 70.17, adjusted *p* < 0.0001), public (mean rank difference = 57.38, adjusted *p* < 0.0001), and academic (mean rank difference = 43.33, adjusted *p* = 0.0009; Figure 3) sector groups. However, there were no significant differences among the four sector groups for graduate-level statements in Round 1 (Figure 4).

#### 3.2.2 Delphi Round 2

Twenty-eight of 29 panellists (96.6%) completed Round 2. We distributed 20 revised statements: 13 at the undergraduate level and seven at the graduate level. Before distribution, we made two adjustments to the undergraduate statements based on panellist feedback from Round 1. We separated one statement on ADME (Absorption, Distribution, Metabolism, and Excretion) concepts into two discrete statements: one addressing knowledge of ADME processes (K9A) and one addressing the factors that affect them (K9B) (Figure 5; Table 3). We also integrated one statement on performance evaluation and adaptation of learning strategies (S4) into a revised learning strategy statement (S2) to consolidate overlapping content (Figure 5; Table 3). All revised statements were reworded to incorporate the median action verbs matching the Bloom’s taxonomy and Dreyfus model levels indicated by panellist ratings in Round 1.

**FIGURE 5:**
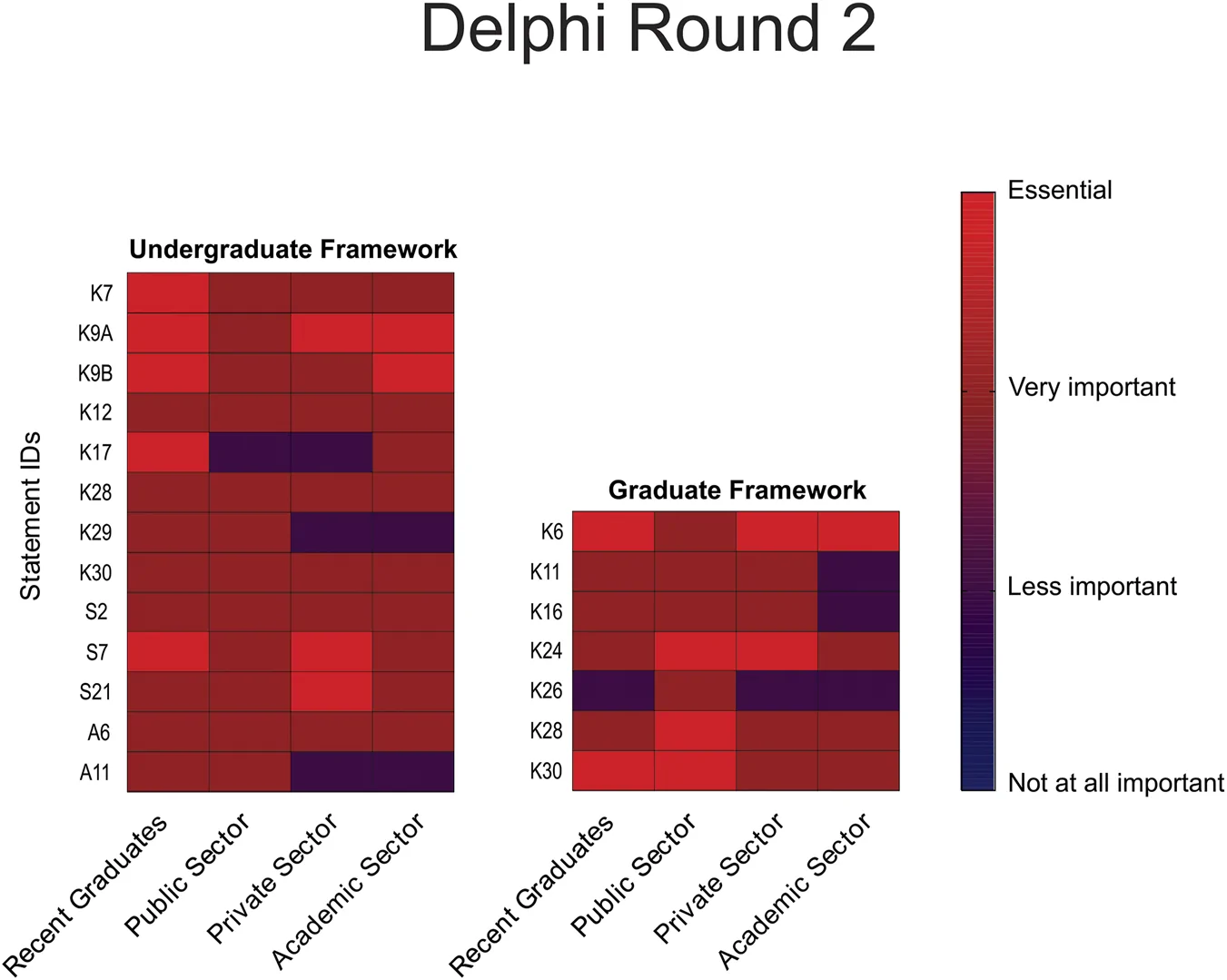
Heatmap of Likert importance ratings for statements re-rated in Delphi Round 2. Undergraduate (n = 13) and graduate (n = 7) framework statements are displayed in separate panels. Columns represent the four Delphi panel groups (Recent Graduates, Public Sector, Private Sector, Academic Sector). Cell color reflects the median rating on a four-point Likert scale ranging from ‘Not at all important’ (dark blue) to ‘Essential’ (bright red), as shown in the color bar.

**TABLE 3:** Round 2 Delphi consensus outcomes for all statements, sorted by academic level.

ID	Statement	Outcome
Undergraduate level (BSc or equivalent)		
K7	Examine the principles of pharmacodynamic drug-drug interactions	Consensus
K9A	Differentiate between the processes of drug absorption, distribution, metabolism, and elimination	Consensus
K9B	Analyze the factors that affect the processes of drug absorption, distribution, metabolism, and elimination	Consensus
K12	Apply knowledge and mechanisms of pharmacokinetic drug-drug interactions to case scenarios	Consensus
K17	Use pharmacogenomic concepts to predict and explain the role of genetic polymorphisms in drug pharmacokinetics and pharmacodynamics, therapeutic outcomes, and adverse drug reactions	No Consensus
K28	Describe the concepts of equity, diversity, inclusion, decolonization, and accessibility in health sciences	No Consensus
K29	Recognize Indigenous traditional medicine and the role of colonialism, racism, and other systemic biases in pharmacology knowledge/practice	No Consensus
K30	Recognize the historical underrepresentation of diverse populations in pharmacology research	Consensus
S2	Plan and implement an effective learning strategy by identifying knowledge gaps, seeking credible resources, and prioritizing actions within defined timelines	Consensus
S7	Plan and conduct database searches (PubMed, Scopus, etc.) and manage citations using reference management tools (Zotero, EndNote, etc)	Consensus
S21	Prepare and communicate effectively in oral presentations by prioritizing key messages and meeting defined time and format standards	Consensus
A6	Self-assess performance and commit to lifelong learning for ethical career development	Consensus
A11	Identify and distinguish between employment opportunities in the pharmacology field and pursue career goals independently	No Consensus
Graduate level (MSc or PhD)		
K6	Evaluate factors influencing drug action, such as lifestyle, genetics, sex, age, formulation, dosage, administration route, and patient adherence	Consensus
K11	Apply advanced pharmacokinetic knowledge in different case scenarios, including population PK, PBPK, multi-compartmental models, and related modelling concepts	Consensus
K16	Apply knowledge of lifestyle and the social determinants of health (diet, access to medications, physical activity) to adjust therapeutic outcomes and anticipate adverse drug reactions	Consensus
K24	Examine the principles of medication safety in different case scenarios to identify hazards, contributing factors, and risk-mitigation strategies	Consensus
K26	Apply principles of equitable access to medicines with regard to affordability, cultural safety, and physically/linguistically accessible information	No Consensus
K28	Analyze how equity, diversity, inclusion, decolonization, and accessibility shape decisions in health sciences, using case examples	Consensus
K30	Apply awareness of historical underrepresentation of diverse populations in pharmacology research to critique studies and adapt evidence use for diverse populations	Consensus

At the undergraduate level, nine of 13 statements reached consensus in Round 2. Four statements did not reach consensus and were excluded. This included a statement on pharmacogenomic concepts (K17), a statement on equity, diversity, inclusion, decolonization, and accessibility in health sciences (K28), and a statement on Indigenous traditional medicine and the role of colonialism and systemic biases in pharmacology (K29; Table 3). A career development statement on identifying employment opportunities (A11) also did not reach consensus and was excluded (Table 3). At the graduate level, six of seven statements reached consensus in Round 2 (Table 3). One statement addressing the application of equitable access principles to medicines (K26) did not reach consensus and was excluded. In contrast to the undergraduate level, the statement addressing equity, diversity, inclusion, decolonization, and accessibility in health sciences (K28) reached consensus among panellists (Table 3).

#### 3.2.3 Final competency frameworks

The Delphi process yielded 27 and 55 consensus statements for undergraduate and graduate levels, respectively. Consensus statements were then rephrased and integrated into competency statements that incorporated the median action verbs corresponding to the level of knowledge or skill acquisition as indicated by panellists in Round 1. Competencies were organized into key and enabling competencies. Key competencies represent high-level performance expectations, and enabling competencies are considered the sub-elements required to achieve them. Some statements were split based on panellists’ feedback, which flagged more than one concept within the same competency or to separate key-level from enabling-level competencies. No competency statements were added or removed. The competency statements were then grouped thematically under six roles expected from the pharmacology graduates: Content Expert, Researcher, Adaptive Learner, Communicator, Collaborator, and Professional. The final undergraduate framework comprised 32 enabling competencies across 12 key competencies, while the final graduate framework comprised 58 enabling competencies across 17 key competencies (Supplementary Files S2 and S3). Both frameworks followed identical architecture to allow a developmental continuum within a shared role-based structure. The final frameworks were reviewed and approved by the panellists.

## 4 Discussion

There is no national consensus on the core competencies for pharmacology graduates in Canada, and this can render graduate competencies dependent on program structure and institutional context (Sinche et al., 2017; Crowley et al., 2025; Guilding et al., 2026). In this study, we employed a mixed methods approach to define core competencies for pharmacology learners at undergraduate and graduate levels. Inquiry with pharmacology professionals from different sectors produced 12 categories of essential knowledge, skills, and attitudes expected of pharmacology learners, which were used to refine and contextualize the stakeholder-amended statement list for Delphi consensus. Across two Delphi rounds, panels of pharmacology professionals and recent graduates reached consensus on a list of 27 and 55 statements for undergraduate and graduate levels, respectively. Post-Delphi reassembly organized these statements into 12 key undergraduate competencies and 17 key graduate competencies. To our knowledge, these represent the first consensus-based, level-specific competency frameworks for pharmacology programs in Canada.

Our study builds on a productive line of international work in pharmacology education. White et al. (2021) first identified 17 core pharmacology concepts using a Delphi approach. Building on this work, the IUPHAR Core Concepts of Pharmacology group identified 24 global core concepts, which were subsequently unpacked with supporting sub-concepts and applied to a concept-based curriculum model (White et al., 2023; Guilding et al., 2024b; 2026). Here, we integrated the 24 core concepts into the knowledge domain statements to ensure that our frameworks reflect internationally validated conceptual content and translate those concepts into undergraduate and graduate performance criteria. The BPS core undergraduate curriculum represents the closest methodological context for situating these framework decisions given the use of the Delphi consensus approach and Knowledge/Skill/Attitude domain organization (Wallace et al., 2021; The British Pharmacological Society, 2026). Interestingly, our undergraduate framework diverges in content scope compared to the BPS curriculum. For instance, pharmacogenomics, translational research, and clinical trial design are listed as core undergraduate content in the BPS curriculum (Wallace et al., 2021; The British Pharmacological Society, 2026). While these domains did emerge as standalone categories from Phase 1 analysis, indicating their importance, none met the consensus threshold required for inclusion in the undergraduate framework. For pharmacogenomics specifically, panellists raised questions about the scope and timing of learning pharmacogenomic concepts, noting that comprehensive coverage may not be necessary before the graduate level. This finding is consistent with recent evidence that pharmacogenomics curriculum implementation remains inconsistent even where formally described as an undergraduate requirement (Harding et al., 2026). Our undergraduate framework instead concentrates on core pharmacokinetic and pharmacodynamic principles and foundational research competencies. This divergence likely reflects the structural diversity of Canadian undergraduate pharmacology programs, which operate across stream-based or interdisciplinary models and differ from the United Kingdom single-honours context for which the BPS curriculum was developed (Wallace et al., 2021; Crowley et al., 2025).

We noted intriguing sector-specific patterns in knowledge or skill area priorities. In the Delphi phase, private sector panellists placed knowledge of pharmacokinetic content below the threshold for the undergraduate level only. Furthermore, private sector panellists reached consensus on advanced pharmacokinetic modelling at the graduate level while the academic sector did not, suggesting distinct expectations about the depth of pharmacokinetic knowledge needed for career readiness. This is in line with the evidence showing that the demand for advanced pharmacokinetic modelling expertise consistently outpaces academic supply, with industry investing substantially in on-the-job development of these competencies at the graduate level (Bonate et al., 2023). On the other hand, academic focus group participants generally prioritized pharmacological knowledge over laboratory practice or technical skills. Likewise, the academic sector panellists ranked the ability to adhere to good laboratory practice below the undergraduate threshold in the Delphi, unlike public and private sector panellists. This may reflect a sector-specific judgment about the appropriate stage of preparation for laboratory competencies. This is consistent with the documented challenges in integrating regulatory competencies such as GLP into academic curricula, despite its importance for professional laboratory practice (Hernandez et al., 2025). A related pattern emerged with recent graduates rating undergraduate statements significantly higher than private, public, and academic panellists, with no corresponding difference at the graduate level. This may reflect the recent graduates’ proximity to the transition into practice by basing the ratings on direct experience of which competencies were needed for the profession. It is also possible that this asymmetry is related to recent graduates’ perceived uncertainty regarding their career destinations compared to other groups, which may have led them to rate a broader range of undergraduate competencies as important. However, this rationale was not directly probed with panellists. Together, these findings suggest that sector-specific expectations may point to areas where academic preparation and workplace demands require closer alignment. Indeed, bridging these gaps will require deliberate curricular decisions about the stage-appropriate integration of technical and applied competencies into pharmacology training.

In recent years, the integration of Equity, Diversity, Inclusion, Decolonization, and Accessibility (EDIDA) principles into health sciences education has been recognized as a priority, given the link between health disparities and inequitable representation in research and clinical care (Tucker and Zecharia, 2022; Jiang et al., 2024; Koenig and Tucker, 2025). In this study, we found that Delphi consensus on equity-related statements varied systematically by how content was categorized within the competency framework. Statements framed as professional skills and attitudes, including cultural comprehension and implicit bias identification, reached undergraduate consensus. In contrast, statements framed as knowledge domains, specifically EDIDA concepts in health sciences, did not reach undergraduate consensus. The graduate-level EDIDA statement, however, reached consensus once reworded to require analytical engagement through case scenarios, an approach panellists described as most appropriate once pharmacological foundations are established. Furthermore, the statement on recognizing historical underrepresentation of diverse populations in research reached consensus at both academic levels when it addressed pharmacology research practices. This pattern suggests that framing of EDIDA content, and the training level at which it is introduced, are both key to its disciplinary application in pharmacology.

A distinct pattern emerged for the knowledge-based statement on Indigenous traditional medicine and systemic biases, which did not reach consensus at either level. Canada’s Truth and Reconciliation Commission Call to Action 24 mandates that medical and nursing schools require coursework on Indigenous health, the history of residential schools, and skills-based training in intercultural competency (Courchene, 2023), yet academic pharmacology programs, which operate outside health professional accreditation frameworks, are not subject to equivalent expectations. This finding points to an unresolved question for the field: how pharmacology programs should engage with Indigenous knowledge, colonial histories, and systemic bias in ways that are discipline-specific, respectful, and educationally meaningful. This is also reflected in the limited published guidance for academic pharmacology curricula in Canada (Leung and Min, 2020; Swidrovich, 2020). Indeed, how pharmacology formalizes this responsibility in competency frameworks warrants further investigation.

Training of MSc and PhD students has largely proceeded by apprenticeship, which influences the quality of training and how graduates are prepared for independent scientific work (Verderame et al., 2018; Morris et al., 2022; ElKhalifa et al., 2024). Adjacent health research fields in Canada, such as health services and policy research, have addressed equivalent gaps by defining doctoral-level competency frameworks (Bornstein et al., 2018; McMahon et al., 2026). These frameworks paired thorough scholarly training with professional and leadership competencies to orient doctoral training toward the diverse academic and non-academic roles (Bornstein et al., 2018; McMahon et al., 2026). Graduate training in pharmacology lacked an equivalent definition, which may relate in part to the absence of profession-specific accreditation mandates and competency standards. Furthermore, the structural heterogeneity of university pharmacology programs has historically complicated the disciplinary consensus required to develop them (Crowley et al., 2025). The graduate framework in this study provides a set of shared minimum competency thresholds that build on the undergraduate framework. For example, the Content Expert role expands to incorporate competencies in advanced pharmacokinetic modelling, the drug discovery and development pipeline, bioinformatics, artificial intelligence, and machine learning. The graduate framework also specifies competencies with no direct undergraduate counterparts. These include innovative problem design and resilience (Adaptive Learner role), scientific leadership and research coordination (Collaborator role), and responsible use of artificial intelligence (Professional role). These additions extend the graduate framework beyond content mastery to the professional and leadership competencies associated with diverse academic and non-academic careers.

It is worth noting that the current graduate framework does not distinguish competency expectations across MSc and PhD levels, which differ in competency emphasis and professional trajectory (ElKhalifa et al., 2024). This designation-specific distinction was challenging to probe given that the majority of participants cited considerable variation in MSc and PhD program structures and research specialties across Canadian universities. Consistent with the framework’s function as a set of shared minimum competency thresholds, the graduate enabling competencies need to be interpreted in light of their specific training contexts at the respective institutions.

The practical utility of our frameworks resides primarily in the enabling competency tier: 32 enabling competencies at the undergraduate level and 58 at the graduate level. Each enabling competency specifies the expected cognitive demand and level of autonomy using Bloom’s taxonomy for knowledge-based statements, and the Dreyfus skill acquisition model for skill-based statements (Anderson and Krathwohl, 2001; Dreyfus, 2004). At the undergraduate level, knowledge-based statements are worded at Bloom’s levels 2 to 4, while skill-based statements carry the qualifier “with guidance as needed” to place expected autonomy at the novice-to-advanced-beginner range on the Dreyfus scale. In the graduate framework, the same content domains advance to Bloom’s levels 5 and 6, while skill-based statements shift to “independently” to signal competent-to-proficient performance on the Dreyfus scale. This progression allows the level-specificity of the frameworks to be observable and actionable at the enabling competency tier.

We primarily foresee the frameworks as curriculum development and mapping instruments. Importantly, the six-role structure provides a practical adoption pathway for pharmacology programs by occupying a deliberate midpoint between IUPHAR concept-based curriculum guides and the entry-to-practice standards for regulated health professionals (White et al., 2023; NAPRA, 2024; RCPSC, 2025; The British Pharmacological Society, 2026), while preparing graduates for the adjacent non-regulated professions they pursue. In addition to supporting curriculum development, we argue that the framework’s primary function is to provide a common ground for institutional quality assurance and program reviews. This allows the design question to shift from “what must programs teach?” to “what should programs collectively commit to as a minimum graduate outcome?” The degree to which this pathway achieves national consistency in pharmacology graduate preparation remains an open and important question for the field.

Several limitations of the present study should be acknowledged. Although we recruited Delphi panellists to reflect the full spectrum of professional and geographical contexts in which pharmacology graduates practice, the panel size of 29 may not fully capture the breadth of institutional perspectives across Canadian programs. Furthermore, the relatively smaller number of non-academic panellists may limit the representativeness of each group within the frameworks. This may be due to time and workload constraints, which are a commonly reported reason for reduced participant engagement across multi-round processes such as Delphi studies (Cunningham et al., 2015; Niederberger et al., 2025). However, the overall panel size (29 in Round 1, 28 in Round 2) falls within the range reported across published Delphi studies and reflects a defensible expert and stakeholder consensus process (Diamond et al., 2014; Belton et al., 2019). Furthermore, it is possible that a portion of Delphi ratings may reinforce views already established during the focus groups and interviews, given that a portion of Delphi panellists participated in both phases of the study. The sector-weighted consensus calculation and the revision of statements across Delphi rounds nonetheless limit the extent of this concern.

In summary, in partnership with the CSPT, we present the first consensus-based competency frameworks for undergraduate and graduate Canadian pharmacology programs, developed from input by multidisciplinary pharmacology professionals. These frameworks address a persistent gap for pharmacology, a non-regulated biomedical discipline, and provide a shared reference for curriculum mapping and program reviews. The frameworks are intended as living documents, designed for iterative revision to maintain relevance, broad representation, and keep pace with advances in the field. Future work will include the development of learning outcomes and tools for program-level assessments mapped onto the enabling competencies. Through ongoing partnership with CSPT and other pharmacology professionals, we will advance the national implementation of these frameworks to provide a shared reference for pharmacology graduate preparation across Canadian programs.

## Data Availability

The original contributions presented in the study are included in the article/Supplementary Material, further inquiries can be directed to the corresponding authors.
