# Supplementary material for: Defining competency frameworks for undergraduate and graduate pharmacology education in Canada
Source: Front Pharmacol. 2026 Oct 28;17:1952861. doi: 10.3389/fphar.2026.1952861 (PMC13752083; doi:10.3389/fphar.2026.1952861)
Supplement: Supplementary file 1 [file Supplementaryfile2.pdf]

Competency statements that met consensus in the Delphi for Undergraduate level (BSc or equivalent)

The following competency categories are integrated and co-developed across the curriculum and are not intended to represent a hierarchy or developmental sequence.

1. Content Expert

Definition: As Content Experts, pharmacology graduates at the BSc level (or equivalent) integrate relevant life science knowledge with pharmacokinetic and pharmacodynamic principles to solve pharmacology problems and predict drug disposition and drug effects.

Graduates are able to:

1.1. Solve problems and explain concepts in pharmacology using knowledge of life science disciplines.
Enabling competency: Analyze the relationships between principles of biochemistry, molecular biology, chemistry, physiology, and pharmacology

1.2. Predict drug disposition within biological systems by examining pharmacokinetic principles.
Enabling competencies:

- Differentiate between the processes of drug absorption, distribution, metabolism, and excretion.
- Explain the factors affecting the processes of drug absorption, distribution, metabolism, and excretion.
- Analyze pharmacokinetic parameters (volume of distribution, bioavailability, steady-state concentration, half-life, clearance, and zero- and first-order kinetics).
- Apply knowledge of pharmacokinetic drug-drug interactions to case scenarios.

1.3. Predict therapeutic and adverse effects by examining pharmacodynamic principles.
Enabling competencies:

- Analyze drug-target interactions and drug selectivity evidence to assess implications for therapeutic and off-target effects.
- Interpret dose/concentration-response relationships to define agonism, antagonism, affinity, potency, efficacy, and therapeutic index.
- Analyze mechanisms of action of drugs used in health and disease and link molecular targets to system-level effects.
- Explain the principles of pharmacodynamic drug-drug interactions.

---

2. Researcher

Definition: As Researchers, pharmacology graduates at the BSc level (or equivalent) use evidence with critical judgment and awareness of bias and limitations. They contribute to generating knowledge through rigorous and well-documented research practice.

Graduates are able to:

2.1. Use pharmacological evidence with critical judgment, scholarly rigour, and awareness of bias and limitations in the evidence base

Enabling competencies:

- Plan and conduct database searches (PubMed, Scopus, etc.) and manage citations using reference management tools (Zotero, EndNote, etc).
- Recognize the historical underrepresentation of diverse populations in pharmacology research and its implications for evidence interpretation.
- Critically evaluate information sources by distinguishing peer-reviewed evidence from non-validated content, including AI-generated content, and by applying explicit quality and validation criteria.

2.2. Perform research-related work efficiently through effective planning and resource management.

Enabling competencies:

- Implement Good Laboratory Practice and adhere to ethical research principles during experimentation, data-handling, and reporting activities.
- Demonstrate attention to detail and quality assurance in research.
- Demonstrate a curious attitude and openness when interpreting data.
- Summarize experimental findings and contextualize them within the literature, with guidance as needed.
- Maintain accurate, organized, and appropriately referenced records in written assignments and laboratory notebooks.

---

3. Adaptive Learner

Definition: As Adaptive Learners, pharmacology graduates at the BSc level (or equivalent) commit to lifelong learning to solve novel problems. They plan and manage their work effectively, meet established timelines, and adjust strategies in response to changing conditions, unexpected outcomes, or unsuccessful approaches.

Graduates are able to:

3.1. Commit to lifelong learning to solve problems in pharmacology using evidence

Enabling competencies:

- Plan and implement an effective learning strategy by identifying knowledge gaps, seeking credible resources, and prioritizing actions within defined timelines.
- Integrate information from multiple sources and prioritize relevant evidence to produce a justified response in different case scenarios.
- Self-assess performance against defined criteria and adapt learning strategies using targeted improvement measures.

3.2. Complete pharmacology-related work in an independent and timely manner.

Enabling competency: Plan and prioritize tasks to meet defined deadlines and manage workload pressure.

3.3. Demonstrate a resilient, adaptable attitude in dealing with failure or unexpected outcomes.
Enabling competencies:

- Regulate responses to failure or unexpected outcomes and maintain professional behaviour.
- Distinguish controllable from uncontrollable factors leading to unexpected outcomes and adapt strategies in response to feedback or new information.

---

4. Communicator

Definition: As Communicators, pharmacology graduates at the BSc level (or equivalent) demonstrate the ability to convey pharmacological concepts clearly and accurately in oral, written, or visual modes. They tailor language and format to the audience and purpose.

Graduates are able to:

4.1. Communicate pharmacological information in an audience-appropriate manner

Enabling competencies:

- Prepare and communicate effectively in oral presentations and reports by prioritizing key messages and meeting defined time and format standards.
- Explain concepts in pharmacology clearly and accurately using terminology appropriate to the audience.

---

5. Collaborator

Definition: As Collaborators, pharmacology graduates at the BSc level (or equivalent) work effectively with diverse partners to achieve shared project goals. They contribute reliably within teams and support inclusive, culturally respectful collaboration.

Graduates are able to:

5.1. Collaborate with diverse partners to deliver pharmacology projects.
Enabling competencies:

- Work effectively and collaboratively within a team to meet shared project goals.
- Recognize, respond to, and respect cultural differences to support inclusive collaboration.

---

6. Professional

Definition: As Professionals, pharmacology graduates at the BSc level (or equivalent) act with integrity, accountability, and equity while pursuing ongoing development.

Graduates are able to:

6.1. Commit to personal, professional development, and ethical working practices.
Enabling competencies:

- Demonstrate professional integrity in pharmacology by acting with honesty, respect, and accountability.
- Identify implicit biases and how to mitigate their influence.
- Support an ethical professional practice while leveraging current and emerging technologies, including artificial intelligence.
- Seek opportunities for personal and professional growth or development.

6.2. Demonstrate an understanding of the societal relevance and impact of pharmacology.
Enabling competency: Describe how medicine use affects society and is shaped by social, cultural, and economic contexts.
