# Supplementary material for: Defining competency frameworks for undergraduate and graduate pharmacology education in Canada
Source: Front Pharmacol. 2026 Oct 28;17:1952861. doi: 10.3389/fphar.2026.1952861 (PMC13752083; doi:10.3389/fphar.2026.1952861)
Supplement: Supplementary file 2 [file Supplementaryfile3.pdf]

Competency statements that met consensus in the Delphi for graduate level (MSc or PhD)

The following competency categories are integrated and co-developed across the curriculum and are not intended to represent a hierarchy or developmental sequence.

1. Content Expert

Definition: As Content Experts, pharmacology graduates at MSc or PhD levels construct, apply, and evaluate pharmacokinetic and pharmacodynamic models by integrating advanced pharmacology knowledge with relevant life science principles.

Graduates are able to:

1.1. Generate knowledge in pharmacology using principles from life science disciplines.
Enabling competencies:

- Develop hypotheses that integrate principles of biochemistry, molecular biology, chemistry, and physiology to explain the biochemical and physiological actions of drugs.
- Select, apply, and justify appropriate mathematical concepts and statistical approaches to evaluate pharmacokinetic and pharmacodynamic datasets.

1.2. Evaluate pharmacokinetic data, methods, and models to assess drug exposure, disposition, and interaction potential.
Enabling competencies:

- Evaluate the factors influencing drug absorption, distribution, metabolism, and excretion using evidence-based information.
- Evaluate the following pharmacokinetic parameters for implications in exposure and dosing: volume of distribution, bioavailability, steady-state concentration, half-life, clearance, and zero- and first-order kinetics.
- Evaluate and predict pharmacokinetic drug-drug interactions using primary evidence.
- Distinguish the methods used in assessing drug pharmacokineticsby parameters measured, assumptions, and main limitations.
- Apply advanced pharmacokinetic knowledge in different case scenarios, including population PK, PBPK, multi-compartmental models, and related modelling concepts.

1.3. Evaluate and construct pharmacodynamic models and mechanistic explanations to predict drug actions, interactions, and variability.
Enabling competencies:

- Construct dose/concentration-response models to derive drug affinity, potency, efficacy, and therapeutic index.
- Evaluate proposed mechanisms of action of drugs used in health and disease using evidence-based information.
- Validate drug-target selectivity evidence to assess on-target or off-target effects.
- Evaluate and predict pharmacodynamic drug-drug interactions based on their mechanisms.
- Evaluate factors influencing drug action, such as lifestyle, genetics, sex, age, formulation, dosage, administration route, and patient adherence.
- Distinguish the methods used in assessing drug pharmacodynamicsby parameters measured, assumptions, and main limitations.

1.4. Evaluate evidence to predict therapeutic outcomes and adverse drug reactions.
Enabling competencies:

- Evaluate how variation in pharmacokinetic and pharmacodynamic parameters drives differences in therapeutic outcomes and adverse drug reactions in case scenarios.
- Deduce the role of genetic polymorphisms in drug pharmacokinetics and pharmacodynamics, therapeutic outcomes, and adverse drug reactions.
- Examine the principles of medication safety in different case scenarios to identify hazards and risk-mitigation strategies.
- When applicable, apply knowledge of lifestyle and the social determinants of health (diet, access to medications, physical activity) to adjust therapeutic outcomes and anticipate adverse drug reactions.

1.5. Use pharmacology evidence to justify decisions on advancing drug candidates from target selection to preclinical and clinical development.
Enabling competencies:

- Distinguish between the different stages of drug discovery and development and explain the associated challenges in each stage.
- Analyze clinical, pre-clinical, and translational research designs for rigor, ethics, implementation considerations, and reporting quality.
- Evaluate how pathophysiological mechanisms yield insights into novel molecular drug targets.
- Analyze applications and limitations of emerging technologies in drug discovery and development (gene modification, bioinformatics, machine learning, artificial intelligence).

1.6. Analyze how equity, diversity, inclusion, decolonization, and accessibility (EDIDA) shape decisions in health sciences, using case examples.
Enabling competency: Examine EDIDA domains in different case scenarios and explain how the identified factors influence the decision points across research, development, and professional contexts.

---

2. Researcher

Definition: As Researchers, pharmacology graduates at MSc or PhD levels critically appraise evidence with explicit attention to bias, limitations, and reproducibility. They formulate and test hypotheses using appropriate methods, efficient planning, and clear documentation. They synthesize findings to produce defensible conclusions that inform decisions.

Graduates are able to:

2.1. Use pharmacological evidence with critical judgment, scholarly rigor, and awareness of bias and limitations in the evidence base
Enabling competencies:

- Apply knowledge of historical underrepresentation of diverse populations in pharmacology research to critique studies and adapt evidence use for diverse populations.
- Conduct reproducible literature searches using primary databases (PubMed, Scopus) and manage citations proficiently using reference management tools(Zotero, EndNote).

- Critically evaluate information sources by distinguishing peer-reviewed evidence from non-validated content, including artificial intelligence-generated content, and by applying explicit quality and validation criteria.
- Critically evaluate study designs and methodologies for validity and bias, including equity and inclusion considerations in population datasets.

2.2. Formulate and test pharmacology hypotheses
Enabling competencies:
- Conduct evidence-based investigations by specifying testable hypotheses, adapting methods to questions, and selecting appropriate analytic approaches.
- Justify methodological decisions in the study design using explicit criteria, for example, validity, feasibility, ethics, bias risk, and reproducibility.

2.3. Design and execute research through efficient planning and resource management.
Enabling competencies:
- Select drugs as pharmacological tools to investigate biological mechanisms, with a justified choice of target, dose, and controls.
- Implement Good Laboratory Practice and ethical research principles across experimental work, data management, and reporting.
- Perform core laboratory techniques with precision and accuracy, meeting predefined quality-control expectations.
- Select appropriate quantitative and qualitative methods for data collection and presentation.
- Demonstrate attention to detail and quality assurance in research.
- Maintain accurate and appropriately referenced records by prioritizing critical information for recording and addressing any inaccuracies.
- Allocates time and materials efficiently by minimizing waste and redundancy across all phases of research.

2.4. Interpret datasets coherently using appropriate statistics and literature synthesis.
Enabling competencies:
- Analyze data, including large datasets, using appropriate statistical approaches, with justification of assumptions and limitations.
- Demonstrate a curious attitude and openness when interpreting data.
- Summarize experimental findings and contextualize them within the literature to produce coherent interpretations.

---

3. Adaptive Learner

Definition: As Adaptive Learners, pharmacology graduates at MSc or PhD levels sustain reflective, lifelong learning to solve complex and evolving pharmacology problems.

Graduates are able to:
3.1. Commit to reflective, lifelong learning to solve complex problems in pharmacology
Enabling competencies:
- Independently plan and prioritize tasks to meet competing deadlines and effectively manage workload pressure.
- Independently access credible resources, identify knowledge gaps, and implement effective learning strategies with defined goals and timelines.

- Integrate information from multiple sources and explicitly weigh evidence quality to produce context-appropriate responses.
- Self-assess performance against rubric criteria and adapt learning plan with justified strategy adjustments in response to formative feedback.

3.2. Design innovative approaches to address complex pharmacology problems

- Define the pharmacology problem by stating assumptions and constraints.
- Develop and select an innovative approach by comparing options against feasibility criteria, for example, resources, timelines, and technical requirements.
- Manage risks and estimate impact by planning mitigations and specifying measurable outcomes.

3.3 Demonstrate a resilient and adaptable attitude in dealing with failure or unexpected outcomes.
Enabling competencies:

- Regulate responses to failure or unexpected outcomes and maintain professional behaviour.
- Distinguish controllable from uncontrollable factors leading to unexpected outcomes and adapt strategies in response to feedback or new information.

---

4. Communicator

Definition: As Communicators, pharmacology graduates at MSc or PhD levels translate pharmacology evidence into clear, accurate messages in oral, written, or visual modes. They tailor content, level of detail, and accessibility to the audience and purpose.

Graduates are able to:

4.1 Communicate pharmacological information in an audience-appropriate manner.
Enabling competencies:

- Prepare and communicate effectively in oral presentations by independently prioritizing key messages and meeting defined time and format standards.
- Write effectively, accurately, and accessibly for both scientific and lay audiences.

---

5. Collaborator

Definition: As Collaborators, pharmacology graduates at MSc or PhD levels work effectively with diverse partners to deliver pharmacology projects. They define roles, coordinate work, and use feedback to sustain productive team function and support culturally respectful and inclusive collaboration.

Graduates are able to:

5.1. Collaborate with diverse partners to deliver pharmacology projects.
Enabling competencies:

- Build and maintain effective working relationships and collaborations through clear role definition and effective communication.

- Demonstrate leadership skills by taking initiative, coordinating tasks, and acting on feedback to improve performance.
- Recognize, respond to, and respect cultural differences to support inclusive collaboration.

---

6. Professional

Definition: As Professionals, pharmacology graduates at MSc or PhD levels act with integrity, accountability, and equity in their professional practice. They apply awareness of the societal impact of pharmacology to their professional judgment.

Graduates are able to:

6.1. Demonstrate ethical, equitable, and responsible professional practice.
Enabling competencies:

- Demonstrate professional integrity in pharmacology by acting with honesty, respect, and accountability.
- Identify and mitigate the influence of implicit biases.
- Promote an ethical professional practice while leveraging current and emerging technologies, including artificial intelligence.
- Seek opportunities for personal and professional growth or development.

6.2. Demonstrate an understanding of the societal relevance and impact of pharmacology.
Enabling competencies:

- Describe how medicine use both affects society and is shaped by social, cultural, and economic factors.
- Distinguish between various employment opportunities in the field of pharmacology and independently pursue career goals.
