# Supplementary material for: Defining competency frameworks for undergraduate and graduate pharmacology education in Canada
Source: Front Pharmacol. 2026 Oct 28;17:1952861. doi: 10.3389/fphar.2026.1952861 (PMC13752083; doi:10.3389/fphar.2026.1952861)
Supplement: Supplementary file 3 [file Table1.docx]

**Table S1.** Research instruments used in the study

| **Phase 1: Focus groups and interviews** | |
| --- | --- |
| 1.1 | Please think of a minimum of 2-3 core knowledge, skills, or attitudes that you believe are essential for pharmacology students to possess at each of the undergraduate, MSc/PhD levels, or both |
| 1.2 | Please review the proposed core competency framework:   - Do you agree with it? - What is missing? - Point out competencies for the MSc/PhD level exclusively. |
| **Phase 2: Delphi questionnaire** | |
| **Delphi round 1** | |
| 2.1.1 | How important is it for pharmacology graduates to achieve the ability described in this statement by the end of their program? |
| 2.1.2 | At what level of Bloom’s taxonomy should the graduates be able to demonstrate this competence upon graduation? Select the appropriate Bloom's level for this statement for each BSc (or equivalent) and MSc/PhD levels. |
| 2.1.3 | Using the adapted Dreyfus Model, select the skill acquisition level that the graduates should reach for this competency statement upon graduation (for each BSc and MSc/PhD) |
| 2.1.4 | Please use this space if you have any feedback on this statement (if applicable) |
| **Delphi round 2** | |
| 2.2.1 | How important is it for pharmacology graduates to achieve the ability described in this statement by the end of their BSc (or equivalent) program? |
| 2.2.2 | How important is it for pharmacology graduates to achieve the ability described in this statement by the end of their MSc or PhD program? |
| 2.2.3 | Please use this space for any feedback on this statement, including the selected verb(s) |

**Table S2.** Categories identified by inductive content analysis of the focus group and interview transcripts.

| **#** | **Category** | **Subcategory** |
| --- | --- | --- |
| **1** | **Data Analysis**  **and**  **Interpretation** | Clinical trial data analysis and interpretation |
|  |  | Data analysis |
|  |  | Big data and omics |
|  |  | Statistical reasoning in clinical trials |
|  |  | Core statistics |
|  |  | Understanding and interpreting data |
| **2** | **Drug Action**  **and**  **Therapeutic Response**  **(PK, PD, and PGx)** | Appreciation of why drugs do not always work |
|  |  | Drug stability |
|  |  | Drug-drug interactions |
|  |  | Factors of drug response variability |
|  |  | Determining PK and PD parameters |
|  |  | Impact of formulation and excipients on bioavailability |
|  |  | Linking PK-PD knowledge across different contexts |
|  |  | Pharmacodynamics (PD) |
|  |  | Pharmacokinetics (PK) |
|  |  | Predicting drug metabolism from chemical structure |
|  |  | Pharmacogenomics (PGx) and drug response variability |
|  |  | Pharmacometrics / PK-PD modelling |
| **3** | **Drug Discovery, Development, and Regulation** | Analytical knowledge of drug development cycle and evaluation |
|  |  | Clinical trial design |
|  |  | Critical thinking in target validation |
|  |  | Current technologies in drug development |
|  |  | Development and validation of drug targets |
|  |  | Drug development processes |
|  |  | Regulatory aspects of drug development |
| **4** | **Interpersonal Skills** | Collaboration and interdisciplinarity* |
|  |  | Communication |
|  |  | Curiosity and proactive attitude |
|  |  | Flexible attitude |
|  |  | Independence |
|  |  | Organisation |
|  |  | Broad soft and interpersonal skills |
|  |  | Teamwork |
| **5** | **Methodological Approaches**  **and**  **Technical Skills** | Learning technical and laboratory skills |
|  |  | Documentation practices |
|  |  | Good Manufacturing Practice (GMP) |
|  |  | Developing and validating assays |
|  |  | Execution of tests and troubleshooting |
|  |  | Good Laboratory Practice (GLP) |
|  |  | Molecular biology assays |
|  |  | Comparing in vivo and in vitro methods |
| **6** | **Principles of Drug Safety** | Adverse drug effects |
|  |  | Drug toxicity |
|  |  | Toxicokinetics |
|  |  | Toxicology |
| **7** | **Principles of Life Sciences** | Cellular physiology and cell biochemistry |
|  |  | Concentration-dilution mathematics |
|  |  | Medicinal chemistry |
|  |  | Physiology and pathophysiology |
|  |  | Cell biology and Anatomy |
| **8** | **Professional Identity and**  **Development** | Adaptation to professional role |
|  |  | Career paths in pharmacology |
|  |  | Ethics |
|  |  | Leadership |
|  |  | Open-mindedness and continuous learning |
|  |  | Adapting knowledge to context |
|  |  | Mentorship |
|  |  | Professional behaviour |
|  |  | Project and budget management |
| **9** | **Scientific Literacy Skills** | Critical evaluation of clinical trial design |
|  |  | Critical evaluation of information |
|  |  | Critical thinking |
|  |  | Critical evaluation of literature |
| **10** | **Scientific Method** | Experimental design |
|  |  | Hypothesis development and testing |
|  |  | Peer-reviewed achievements to demonstrate pharmacology knowledge |
|  |  | Diverse toolkit to interrogate scientific questions |
| **11** | **Translational**  **and**  **Clinical Pharmacology** | Application of pharmacology skills |
|  |  | Connection between fundamentals and clinical practice |
|  |  | Describing PK parameters in clinical settings |
|  |  | Extrapolating data from animals to humans |
|  |  | Translation and clinical relevance of studies |
| **12** | **Emerging**  **or**  **Specialized Themes** | Biotherapeutics |
|  |  | Drug toxicology, health outcomes, and environmental link |
|  |  | Emerging technologies in pharmacology |
|  |  | Mental imaging skill (imagination) |
|  |  | Mitigation of drug effects on the environment |
|  |  | New Approach Methodologies (NAMs) |
|  |  | Basic programming skills |
|  |  | Shift from animal models to organoids |

** Collaboration and Interdisciplinarity contains sub-codes: Bridging pharmacology with other disciplines; Interdisciplinary collaboration and communication; Openness to diverse perspectives.*

**Table S3.** Round 1 Delphi consensus outcomes for all statements, sorted by program level.

| **ID** | **Statement** | **Outcome** | |
| --- | --- | --- | --- |
|  |  | **Undergraduate (BSc)** | **Graduate (MSc/PhD)** |
| K1 | Relevant principles in biochemistry, molecular biology, medicinal chemistry, and physiology. | Consensus | Consensus |
| K2 | Mathematical concepts and statistical analyses relevant to pharmacokinetic and pharmacodynamic data. | No consensus | Consensus |
| K3 | Principles of drug-target interaction and drug selectivity. | Consensus | Consensus |
| K4 | Concepts: Dose/concentration-response relationships and the related concepts including agonists and antagonists, drug affinity, drug potency, drug efficacy, and therapeutic index. | Consensus | Consensus |
| K5 | Mechanism of action of drugs that are used in health and disease. | Consensus | Consensus |
| K6 | Factors influencing drug action (lifestyle, genetics, sex, age, formulation, administration route, patient adherence). | No consensus | Revisit in Round 2 |
| K7 | Pharmacodynamic drug-drug interactions. | Revisit in Round 2 | Consensus |
| K8 | Appropriate and evolving methods for investigating drug pharmacodynamics. | No consensus | Consensus |
| K9 | Concepts: Drug absorption, distribution, metabolism, elimination and the factors that affect these processes. | Revisit in Round 2 | Consensus |
| K10 | Concepts: Dose/concentration-time relationships and the related concepts including volume of distribution, bioavailability, steady-state concentration, drug half-life, drug clearance, and zero- and first-order kinetics. | Consensus | Consensus |
| K11 | Advanced pharmacokinetics processes such as population PK, PBPK, multi-compartmental models, modelling and simulation concepts. | No consensus | Revisit |
| K12 | Pharmacokinetic drug-drug interactions. | Revisit in Round 2 | Consensus |
| K13 | Appropriate and evolving methods investigating drug pharmacokinetics. | No consensus | Consensus |
| K14 | Role of pharmacokinetics and pharmacodynamics in drug response variation. | No consensus | Consensus |
| K15 | Role of occupational exposures in therapeutic outcomes and adverse drug reactions. | No consensus | No consensus |
| K16 | Role of lifestyle and the social determinants of health therapeutic outcomes and adverse drug reactions (diet, access to medications, physical activity). | No consensus | Revisit in Round 2 |
| K17 | Role of genetic polymorphisms on pharmacokinetics, pharmacodynamics, therapeutic outcomes, and adverse drug reactions. | Revisit in Round 2 | Consensus |
| K18 | Principles of pharmacogenomics explaining individual variations in drug response (e.g. epigenetics of generational trauma). | No consensus | No consensus |
| K19 | Stages and challenges of drug discovery and development. | No consensus | Consensus |
| K20 | The role of structure-activity relationships in drug design and drug optimization. | No consensus | No consensus |
| K21 | Pathophysiology as a basis for identifying new drug targets. | No consensus | Consensus |
| K22 | Principles of clinical, pre-clinical, and translational research (design, implementation, reporting, ethics). | No consensus | Consensus |
| K23 | Emerging technologies in drug discovery and development (gene modification, bioinformatics, machine learning, and AI). | No consensus | Consensus |
| K24 | Principles of medication safety. | No consensus | Revisit in Round 2 |
| K25 | Principles of toxicology. | No consensus | No consensus |
| K26 | Principles of equitable access to medicines (affordability, cultural safety, and physically/linguistically accessible information). | No consensus | Revisit in Round 2 |
| K27 | Sustainability in pharmacology (pharmaceutical industry environmental impact, eco-friendly practices). | No consensus | No consensus |
| K28 | Concepts of equity, diversity, inclusion, decolonization, and accessibility in health sciences. | Revisit in Round 2 | Revisit in Round 2 |
| K29 | Indigenous traditional medicine and role of colonialism, racism, and other systemic biases in pharmacology knowledge/practice. | Revisit in Round 2 | No consensus |
| K30 | Knowledge of historical underrepresentation of diverse populations in pharmacology research. | Revisit in Round 2 | Revisit in Round 2 |
|  |  |  |  |
| S1 | Work to deadlines and effectively manage time and pressure. | Consensus | Consensus |
| S2 | Identify and access resources, identify gaps in knowledge, and develop effective learning strategies. | Revisit in Round 2 | Consensus |
| S3 | Integrate information from a range of sources and apply knowledge to different scenarios. | Consensus | Consensus |
| S4 | Evaluate performance and adapt learning strategies. | Revisit in Round 2 | Consensus |
| S5 | Demonstrate ability to apply creative and innovative approaches in complex, real-world settings. | No consensus | Consensus |
| S6 | Critical reassessment of the current and past research with particular attention to the inclusion and representation of Indigenous and other underrepresented communities. | No consensus | No consensus |
| S7 | Demonstrate the ability to use primary literature and databases (PubMed, Scopus, etc.) and reference manager tools (Zotero, Endnote, etc). | Revisit in Round 2 | Consensus |
| S8 | Demonstrate the ability to critically evaluate information from multiple sources (peer-reviewed journals vs. non-validated content, including AI-generated). | Consensus | Consensus |
| S9 | Demonstrate the ability to critically evaluate study design and methodologies, including biases in datasets (e.g. assessment of equity and inclusion in population studies). | No consensus | Consensus |
| S10 | Demonstrate the ability to employ evidence-based, problem-solving approach (hypothesis formulation and testing, design, and analysis using qualitative and quantitative tools). | No consensus | Consensus |
| S11 | Demonstrate the ability to make appropriate decisions about methodology in study design. | No consensus | Consensus |
| S12 | Demonstrate the ability to employ drugs as pharmacological tools in scientific research. | No consensus | Consensus |
| S13 | Demonstrate the ability to use quantitative and qualitative methods to collect, process and present data. | No consensus | Consensus |
| S14 | Demonstrate the ability to be precise and accurate in core lab skills. | No consensus | Consensus |
| S15 | Demonstrate the ability to adhere to Good Laboratory Practice and ethical research principles. | Consensus | Consensus |
| S16 | Demonstrate the ability to accurately record, organize, and reference information (written assignments and lab books). | Consensus | Consensus |
| S17 | Demonstrate the ability to select and interpret the appropriate statistical approach. | No consensus | Consensus |
| S18 | Demonstrate the ability to analyze and interpret large datasets. | No consensus | Consensus |
| S19 | Demonstrate the ability to summarize experimental findings and contextualize them within the literature. | Consensus | Consensus |
| S20 | Demonstrate the ability to write effectively and accessibly for both scientific and lay audiences. | No consensus | Consensus |
| S21 | Demonstrate the ability to communicate effectively in oral presentations. | Revisit in Round 2 | Consensus |
| S22 | Demonstrate the ability to identify and maintain effective working relationships and collaborations. | No consensus | Consensus |
|  |  |  |  |
| A1 | Resilience, adaptability, and courage to uphold principles under pressure or when facing failure or unexpected outcomes. | Consensus | Consensus |
| A2 | Attention to detail, quality assurance in research. | Consensus | Consensus |
| A3 | Resource-efficient, environmentally-conscious, and supportive research practices. | No consensus | Consensus |
| A4 | Curiosity and openness in data analysis and interpretation. | Consensus | Consensus |
| A5 | Cultural comprehension, responsiveness, and respect. | Consensus | Consensus |
| A6 | Self-assess performance and commit to lifelong learning for ethical career development. | Revisit in Round 2 | Consensus |
| A7 | Implicit bias identification and mitigation. | Consensus | Consensus |
| A8 | Use of current and emerging technologies (artificial Intelligence) for ethical career development practice. | Consensus | Consensus |
| A9 | Practice and recognize leadership qualities. | No consensus | Consensus |
| A10 | Appreciation for societal relevance and impact of pharmacology. | Consensus | Consensus |
| A11 | Identification and distinguishing between employment opportunities in the field and independent career goal pursuit. | Revisit in Round 2 | Consensus |
